# Data and methods for assessing urban green infrastructure using GIS: A systematic review

**DOI:** 10.1371/journal.pone.0324906

**Published:** 2025-06-11

**Authors:** Xiaomian Wu, Jianquan Liu, Yukun Hou

**Affiliations:** 1 College of Art, Suzhou University of Science and Technology, Suzhou, China; 2 Shanghai Academy of Fine Arts, Shanghai University, Shanghai, China; Peking University, CHINA

## Abstract

Comprehensive and visual assessments utilizing Geographic Information Systems (GIS) offer an empirical foundation for the planning, construction, and optimization of Urban Green Infrastructure (UGI), effectively promoting its sustainable development. A comprehensive review of this field clarifies the research methods, application scope, trends, and challenges associated with using GIS to advance UGI development. This study synthesizes research findings from the Science Citation Index (SCI) and Social Science Citation Index (SSCI) within the Web of Science (WOS) database, as well as from the Scopus database, for the period from January 1, 2020, to June 30, 2024. The initial dataset included 640 articles from WOS and 952 articles from Scopus. After removing 1,572 duplicates and irrelevant studies, the final selection consisted of 20 articles. The integration of both WOS and Scopus databases ensures a comprehensive capture of current trends and limitations in GIS-based UGI assessments. This study centers on the scope, data sources, theoretical models, analyses, and objectives of GIS-based UGI assessments. The research indicates that over the past five years, GIS-based UGI assessments have primarily focused on areas such as accessibility, ecosystem service potential, resilience, and environmental justice, in addition to non-ecological aspects such as social benefits and aesthetics. While the integration of diverse data and analytical indicators into GIS has enhanced assessment comprehensiveness, and AI technologies have deepened data analysis, field research with urban residents remains crucial, underscoring the importance of inclusiveness in the study. This study also reveals a significant increase in interdisciplinarity in GIS-based assessments of UGI. The integration of assessment methods from ecology, computer science, urban planning, sociology, aesthetics, and other disciplines demonstrates that research in this field has fully considered ecological, social, economic, and humanistic factors, thereby more comprehensively reflecting the integrated needs of sustainable urban development.

## Introduction

The United Nations Sustainable Development Goals (SDGs) were introduced in September 2015, one of which is SDG11: Sustainable Cities and Communities, with a specific focus on urban areas [1]. SDG11 acknowledges the remarkable increase in urbanization worldwide and the significance of this phenomenon for human welfare and its crucial role in promoting global sustainable development [2]. In view of the contradictions and conflicts between socioeconomic development and natural ecosystems [3], concepts such as sustainable development and circular economy have become the core of current environmental issues [4–7]. Green infrastructure (GI) has emerged as one of the key strategies for achieving sustainable development as an effective way to reconcile environmental, social, and economic development [8–10]. The notion of GI plays a pivotal role in the investigation of the harmonious coexistence between human beings and nature [11], and the utilization of GI is seen as essential for sustainability [12]. Presently, both industrialized and developing nations are advocating for the implementation of environmentally-friendly infrastructure in urban areas. [13] stated that the emphasis on the design, planning, and administration of GI is frequently placed on cultural and ecological services, such as aesthetics, recreation, and cultural heritage. GI has the ability to promote good health and well-being [14–16], safeguard and improve urban biodiversity [17], and contribute to a high-quality built environment in order to achieve environmental sustainability [18]. The character of GI is defined by several principles, as revealed by various studies. These principles include: 1) the incorporation of both urban and rural areas and environments in a cohesive manner, 2) seamless integration with other urban infrastructures, 3) the ability to provide a diverse range of services through multifunctionality, 4) the establishment of connectivity between the form and function of the landscape, 5) the consideration of nature and cultural processes at multiple scales, and 6) the utilization of interdisciplinary approaches that combine the expertise of different disciplines [19].

Simultaneously, as society rapidly advances, the global proliferation of GI plans is on the rise, indicating the growing significance of environmental protection and sustainable development to human society [20] . Nevertheless, it is important to acknowledge that, despite the growing significance of GI, there remains a lack of widespread agreement over the precise definition and scientific categorization of GI within both academic and practical spheres. The current situation to some degree limits the successful advancement of GI planning and the creation of consistent standards. Moreover, while numerous studies have examined the fundamental principles [21,22] that should be adhered to in environmental infrastructure planning, these principles often have a theoretical bias and lack comprehensive and practical guidance for the execution and implementation of GI in real spatial planning. The area of urban planning is presently experiencing unparalleled transformations. The quick iteration of planning methodologies and the continual innovation of decision-making process implementation, including the utilization of big data, Artificial Intelligence (AI), and other modern technologies, have presented novel prospects for urban planning, along with accompanying difficulties. Specifically, challenges such as ongoing population expansion, deterioration of the environment, and economic reorganization are increasing the requirements for geographic information planning. This necessitates a reassessment of the suitability and efficiency of current planning frameworks.

Quantitative analyses based on Geographic Information Systems (GIS) are increasingly important for evaluating urban green infrastructure (UGI). With the help of GIS, researchers can leverage a wide range of remote sensing (RS) datasets and analyze them across various spatial scales [23]. This multi-scale spatial analysis method helps reveal the performance of UGI under various environmental conditions. After inputting spatial data, such as urban neighborhoods, path raster images, and green space layers, into GIS and combining them with data on local residents’ preferences for green space accessibility obtained through questionnaire surveys, researchers can quantitatively assess the accessibility and quality of urban green spaces [24]. GIS enables researchers to analyze not only the spatial vulnerability of GI to flooding but also the social vulnerability associated with socio-economic characteristics and behavioral responses to disasters [25]. Through this type of analysis, key areas of vulnerability can be identified, and targeted GI solutions can be proposed to enhance resilience to natural disasters, such as flooding. Moreover, by integrating the BREEAM-CM assessment criteria with GIS analysis, researchers can evaluate the sustainability performance of different urban sub-regions, thereby assisting local governments in prioritizing areas for intervention [26]. Nowadays, GIS-based analysis of UGI not only provides a scientific assessment tool for sustainable urban development but also offers an empirical basis for policy formulation and implementation through the visualization of analysis results.

A Several research outcomes on the use of GIS for assessing UGI are now publicly available. Although these outcomes are systematic reviews conducted using the PRISMA standard, they fail to comprehensively reveal the objectives, methods, and comprehensive development of GIS-based assessments of UGI in recent years due to the lack of focus on UGI or insufficiently comprehensive assessment content. [27] conducted a comprehensive review of RS research on urban ecosystem services (UES) between 2010 and 2020, offering a macroscopic reference for the progress of UES research within the context of GIS applications. However, UES and UGI differ significantly in conceptual scope, planning and management, and implementation priorities. Moreover, the literature reviewed, which spans from 2010 to 2020, does not reflect the developments and changes in the use of GIS for assessing urban green spaces after 2020. Reference [28] has addressed how GIS can be used for effective asset and disaster risk management in the context of hazards that cities and communities may face. Nevertheless, the research results did not include a review of the accessibility and aesthetic assessment of urban green spaces or UGI. [29] addressed the fragmentation of landscapes triggered by urbanization, reviewed the roles and values of multiple tools and resources in the field of GIS for understanding and optimizing ecological networks and green spaces. The results of this research were predominantly focused on the ecological and biological dimensions of assessment, thereby neglecting the social benefits and aesthetic assessment of urban green spaces. [30] aimed to revitalize urban vacant land from an ecological perspective. Their systematic review of the literature on planning tools for urban vacant land revitalization highlights the importance of GIS in assessing and revitalizing urban vacant land. This research is not grounded in the theoretical framework of ecological function and does not directly address social benefits or aesthetic assessment. Consequently, recent studies using GIS to assess UGI are limited by a narrow research perspective and singular research objectives, failing to provide a comprehensive and systematic overview of the current research status in this field.

Based on this key observation, this study revisits the comprehensive assessment of UGI and attempts to provide a more scientific overview of the GIS tools and techniques used in assessing UGI. Although our study is not limited to a specific technological approach, we focus on the application of GIS in the field. Therefore, the objectives of this systematic review are to:

- Sort out which aspects of UGI can be assessed by GIS. The research and practice related to UGI encompass a broad range of areas, including accessibility, aesthetic evaluation, natural disaster defence, improvement of urban residents’ quality of life, enhancement of social benefits, and improvement of the natural environment;
- How to use GIS for assessment. This review focuses on the sources of data, the way of data fusion, the use of models and theories, and the way of validation in the assessment of UGI using GIS;
- Advances in assessment methods. This review will also examine the changes and opportunities that the intervention of new hardware devices and AI algorithms has brought to the assessment of UGI using GIS.

The academic contribution of this review is to sort out the application areas of GIS in UGI assessment, the implementation methods of GIS assessment, and the development and innovation of assessment methods.

The paper is organized as follows: The Introduction section provides an overview of the current state of research on the assessment of UGI and offers a useful comparison with the scope and focus of our study. The Materials and Methods section details the methodology and material preparation used in this systematic literature review. The Results section provides an overview of the research object and reports on the characteristics of the journals and the specific application of GIS in GI assessment. Based on the results of the study, the Discussion section explores the three questions posed in this paper, focusing on the scope, value, future trends of GIS application in UGI assessment, and the limitations of the study. Finally, the Conclusion section outlines the practical implications of this research, while also indicating potential avenues for future inquiry.

## Materials and methods

### Data sources and search strategy

A thorough systematic review was conducted following the requirements of the Preferred Reporting Items for Systematic Reviews and Meta-Analyses (PRISMA) [31]. The search strategy for this paper included the use of a combination of keywords related to “GIS” and “UGI” from a variety of sources. We conducted a search that included journals published between 1 January 2020 and 30 June 2024, using the Science Citation Index (SCI) and Social Science Citation Index (SSCI) within the Web of Science (WOS), as well as the Scopus database. Scopus and WOS are two pivotal databases in the academic landscape. They encompass a broad array of scientific disciplines and compile a vast repository of peer-reviewed publications that meet stringent quality criteria. Researchers can leverage these platforms to evaluate the impact of scholarly works, identify groundbreaking research, and refine their search methodologies. These databases provide rich metadata, which is essential for enhancing the visibility and discoverability of research. The established citation standards within these databases are instrumental in assessing research outcomes and promoting global academic collaboration. The utilization of Scopus and WOS has significantly enhanced the accessibility, reliability, and quality of scientific research [32,33]. Consequently, the literature obtained from both databases was meticulously reviewed. The data for this study were extracted on August 4, 2024. We carefully reviewed all relevant studies and selected those that matched our specific criteria for inclusion, which were based on the significance of their title, abstract, or keywords. Table 1 presents a comprehensive list of search phrases.

**Table 1:** List of search terms.

Database or website	Search strategy
SCI and SSCI within the WOS	TS = (“ Green infrastructure*” OR “ green space*” OR “ greenspace*” OR “ green roof*” OR “ greenstreet*”) AND TS = (“ GIS” OR “ Geographic* Information Science*” OR “ Geographic Information Technology” OR “ Geographic Information System” )
Scopus	( ( TITLE-ABS-KEY ( “ Geographic Information System” ) ) OR ( TITLE-ABS-KEY ( “ Geographic Information Technology” ) ) OR ( TITLE-ABS-KEY ( “ Geographic* Information Science*” ) ) OR ( TITLE-ABS-KEY ( “ GIS” ) ) ) AND ( ( TITLE-ABS-KEY ( “ greenstreet*” ) ) OR ( TITLE-ABS-KEY ( “ green roof*” ) ) OR ( TITLE-ABS-KEY ( “ greenspace*” ) ) OR ( TITLE-ABS-KEY ( “ green space*” ) ) OR ( TITLE-ABS-KEY ( “ Green infrastructure*” ) ) )
Date	January 1, 2020 to June 30, 2024

### Screening and eligibility criteria

To ensure comprehensive identification of relevant articles, we supplemented our search with a manual review of the available literature. Two researchers independently screened the titles, abstracts, and full texts of the articles. Titles and abstracts were initially assessed for relevance to the research question, with articles meeting all inclusion criteria selected for full-text review. Any disagreements were resolved through consensus between the two researchers. In cases where consensus could not be reached, the third researcher mediated the discussion to resolve the disagreement.

The inclusion criteria for identifying relevant studies were as follows:

- The article was written in English;
- The article was peer-reviewed;
- The article was published between 1 January 2020 and 30 June 2024;
- The article involved empirical research on UGI, without restrictions on the type or number of GI;
- The article employed GIS in the assessment of UGI.

Exclusion criteria included:

- The article was repetitive or contained redundant information;
- The research subject did not involve GI within an urban context;
- The research focused on UGI projects that had not yet been constructed.

### Quality assessment

To evaluate the quality of the selected articles, we have identified a set of assessment criteria. The following questions were used to guide the assessment process:

- QA1: Were the objectives and research questions clearly specified? [34]
- QA2: Did the article specify the name, version, and developer of the GIS used?
- QA3: Was the research process transparent and reproducible? [34]
- QA4: Did the article provide an empirical assessment of UGI and propose an optimization plan based on the results?

Each question was rated using a three-level scale.

The criteria used for QA1 are:

- **Fully matched** Objectives and research questions were explicitly stated.
- **Partially matched** Objectives were clear but could be improved.
- **Not matched** Objectives were unclear or not related to the research.

The criteria used for QA2 are:

- **Fully matched** Name, version, and developer of the GIS were stated.
- **Partially matched** GIS name was provided, but version and developer were missing
- **Not matched** No details about the GIS were provided.

The criteria used for QA3 are:

- **Fully matched** Methodology, technologies, and data were clearly outlined.
- **Partially matched** Minor details were missing (e.g., datasets not readily available).
- **Not matched** Methodology was insufficient to reproduce the research.

The criteria used for QA4 are:

- **Fully matched** UGI was assessed and an optimization plan was proposed.
- **Partially matched** UGI was assessed but no optimization plan was provided.
- **Not matched** No assessment or optimization plan was presented.

We subsequently evaluated whether the selected papers met the established criteria and, if so, the extent to which they did. Specifically, we assigned a score of 1 to papers that fully met the criteria, 0.5 to those that partially met the criteria, and 0 to those that did not meet the criteria at all. The first and second authors independently evaluated each article. In cases of disagreement, the two authors attempted to reach a consensus through discussion. If consensus could not be reached, the third author mediated the discussion to resolve the disagreement. The period for the assessment of the article quality was from September 12, 2024 to September 21, 2024.

## Results

**Fig 1:**
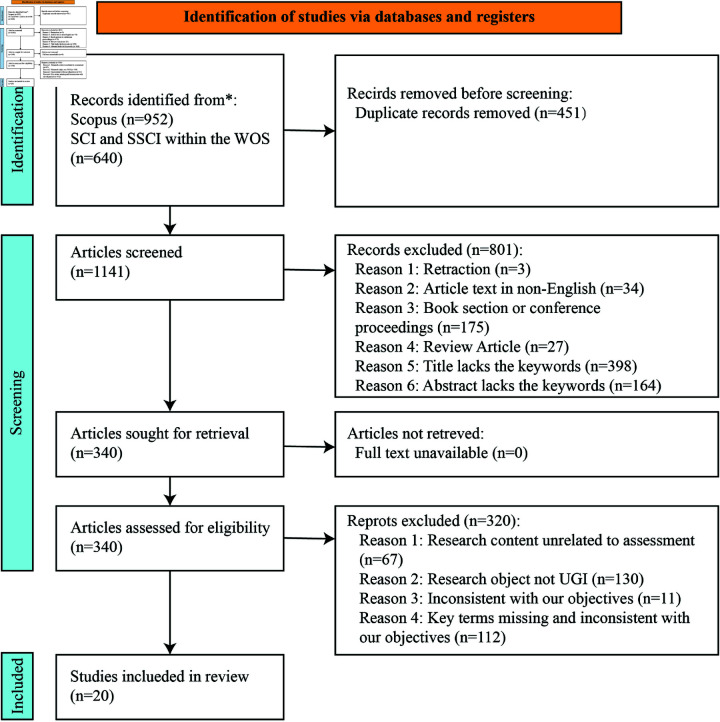
PRISMA diagram.

A comprehensive literature search was conducted through the WOS and Scopus databases, resulting in the retrieval of a total of 1,592 papers. Following the removal of duplicates, 1,141 papers were ultimately retained for further analysis. S1 Table shows the complete list of included and excluded references. During the initial screening stage, the following types of literature were excluded: retracted articles (n = 3); article text in non-English (n = 34); article type of book section or conference proceedings (n = 174); review article (n = 27); titles missing keywords (n = 398); and abstracts missing keywords (n=164). Following the initial screening, a total of 340 articles were advanced to the full-text assessment stage. During the subsequent full-text assessment process, the following categories of articles were excluded: research content unrelated to the assessment (n = 67); research object not UGI (n = 67); inconsistent with our objectives (n = 11); and key terms missing and inconsistent with our objectives (n = 112). S2 Table lists the reasons for each of the excluded papers. Ultimately, a total of 20 articles were included in this study, and the relevant results have been presented in the PRISMA diagram flow in Fig 1. S3 Table gives the PRISMA checklist that was used. The complete list of studies can be seen in Table 2.

**Table 2:** All of the assessed studies in this systematic review.

Title of study	Reference
A GIS assessment of the green space percentage in a big industrial city (Dnipro, Ukraine)	(Buchavyi, Lovynska *et al*. 2023) [35]
A GIS-based analysis of the urban green space accessibility in Craiova city, Romania	(Vîlcea and È˜oÈ(tm)ea 2020) [36]
Accurate suitability evaluation of large-scale roof greening based on RS and GIS methods	(Xu, Luo *et al*. 2020) [37]
Analysis and Optimized Location Selection of Comprehensive Green Space Supply in the Central Urban Area of Hefei Based on GIS	(Huang, Yu *et al*. 2023) [38]
Assessing carbon storage and sequestration benefits of urban greening in Nador City, Morocco, utilizing GIS and the InVEST model	(Rachid, Elmostafa *et al*. 2024) [39]
Assessing green infrastructures using GIS and the multi-criteria deci-sion-making method: the case of the Al baha reGIon (Saudi arabia)	(Mobarak, Shrahily *et al*. 2022) [40]
Enhancing sustainable urban planning through GIS and multiple-criteria de-cision analysis: a case study of green space infrastructure in Taif Province, Saudi Arabia	(Waheeb, Zerouali *et al*. 2023) [41]
Establishment of a geographic information system-based algorithm to ana-lyze suitable locations for green roofs and roadside trees	(Kim, Oh *et al*. 2021) [42]
Evaluate of Green space (Parks) in Duhok city by use Image satellite, Google earth, GIS,(NDVI), and Field survey Techniques	(Mohammed and Hammo 2023) [43]
Evaluation of urban green space per capita with new remote sensing and ge-ographic information system techniques and the importance of urban green space during the COVID-19 pandemic	(Pouya and Aghlmand 2022) [44]
Geographic information system based combined compromise solution (Co-CoSo) method for exploring the spatial justice of accessing urban green spac-es, a comparative study of district 22 of Tehran	(Ghasemi, Behzadfar *et al*. 2022) [45]
GIS modeling of green infrastructure of mediterranean cities for the man-agement of urbanized ecosystems	(Mironova 2021) [46]
GIS-Based Multi-Criteria Analysis for Selecting Suitable Areas for Urban Green Spaces in Abomey-Calavi District, Southern Benin	(Osseni, Dossou-Yovo *et al*. 2023) [47]
Gravity model toolbox: An automated and open-source ArcGIS tool to build and prioritize ecoloGIcal corridors in urban landscapes	(Wanghe, Guo *et al*. 2020) [48]
Green infrastructure design using GIS and spatial analysis: A proposal for the Henares Corridor (Madrid-Guadalajara, Spain)	(Rodríguez-Espinosa, Aguilera-Benavente *et al*. 2020) [49]
Green roof ecosystem services in various urban development types: A case study in Graz, Austria	(Hoeben and Posch 2021) [50]
Research on optimization strategies for urban park green space planning in Nanjing based on GIS from the perspectives of network analysis and Thies-sen polygon theory	(Yang 2024) [51]
Suitable site selection for urban green space development using geographic information system and remote sensing based on multi criterion analysis	(Hailemariam 2021) [52]
Urban green space planning based on remote sensing and geographic infor-mation systems	(Bai, Li *et al*. 2022) [53]
Visual assessment of historic landmarks based on GIS and survey: a study of view and viewing of Tiger Hill in Suzhou, China	(Zhang, Yan *et al*. 2024) [54]

**Fig 2:**
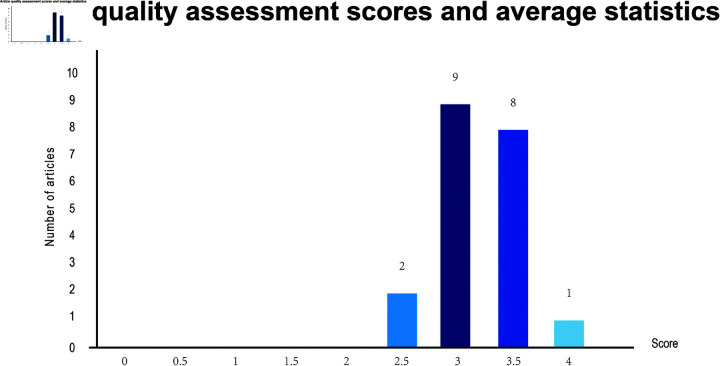
Paper distribution by quality score.

Regarding the quality assessment. The final quality assessment results are summarized in S4 Table. Among the articles we ultimately chose to include, there were many high quality articles, as evidenced by the scores in Fig 2. The quality of the articles was high and the average quality score was 3.2 on a scale of 4.

### Characteristics of selected studies

#### Journals and publishers.

**Fig 3:**
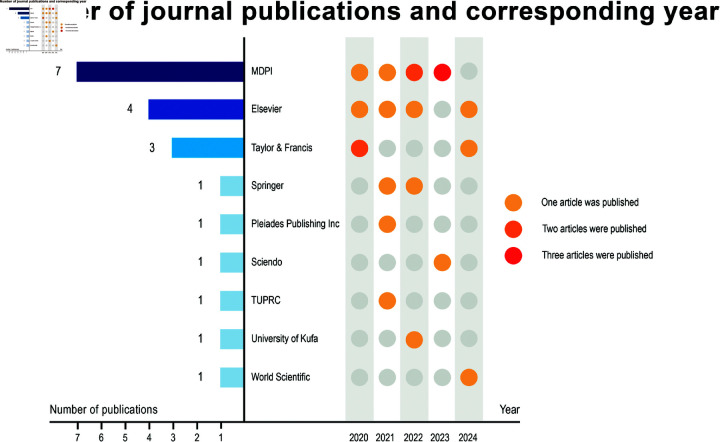
Published publisher and quantity.

The selected papers were published in a total of 20 journals, distributed among the following publishers: MDPI (n = 7), Elsevier (n = 4), Taylor & Francis (n = 3), Springer (n = 1), Pleiades Publishing Inc (n = 1), Sciendo (n = 1), Tehran Urban Research and Planning Center (n = 1), World Scientific (n = 1), and University of Kufa (n = 1), as indicated in Fig 3.

The journals associated with each publisher are as follows:

- **MDPI**: *Buildings*, *Forests*, *Sustainability*, *Water*, *Land*, *Remote Sensing*, and *Applied Sciences*.
- **Elsevier**: *Sustainable Futures*, *Ecological Indicators*, *Urban Forestry & Urban Greening*, and *Global Ecology and Conservation*.
- **Taylor & Francis**: *Geografisk Tidsskrift-Danish Journal of Geography*, *Landscape Research*, and *Journal of Asian Architecture and Building Engineering*.
- **Springer**: *Environmental Monitoring and Assessment*.
- **Pleiades Publishing Inc**: *Arid Ecosystems*.
- **Sciendo**: *Ekológia (Bratislava)*.
- **World Scientific**: *International Journal of Modern Physics C*.
- **Tehran Urban Research and Planning Center (TUPRC)**: *International Journal of Human Capital in Urban Management*.
- **University of Kufa**: *Kufa Journal for Agricultural Sciences*.

#### Location.

**Fig 4:**
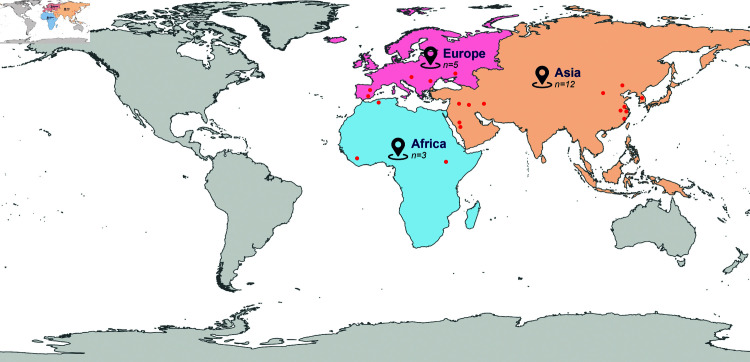
Continents covered by the reviewed articles.

The selected publications span thirteen nations as the sites of the conducted studies. Table 3 details the countries, cities, and specific research locations involved. Notably, the majority of the studies were executed in China (n = 6), Saudi Arabia (n = 2), and Spain (n = 2), with the remaining nations each represented by a single study. In terms of the distribution by continent, the number of studies conducted in Asia was 12, followed by 5 in Europe and 3 in Africa, as illustrated in Fig 4.

**Table 3:** Study cities and sites in selected studies.

Study	Research country	Research City	Specific study site
[35]	Ukraine	Dnipro	Administrative districts, parks, gullies and health protection zones
[36]	Romania	Craiova City	Urban green spaces in Craiova city
[37]	China	Xiamen	Roof Greening on Xiamen Island
[38]	China	Hefei	Park green space in the central area of Hefei City
[39]	Morocco	Nador City	The urban center area and the northern part of the Gourougou forest in its vicinity
[40]	Saudi Arabia	Al-Baha	Various areas in the Al Bahah region, including Al Bahah, Elmandaq, Alatawlah and the central part of Buljurshi
[41]	Saudi Arabia	Taif	Urban green spaces in the Taif region
[42]	Korea	Seoul	Seoul and Surrounding Areas
[43]	Republic of Iraq	Kurdistan Region	Parks in Duhok city
[44]	Türkiye	Malatya	Battalgazi and its various districts in the city of Malatya
[45]	Iran	Tehran	District 22 in Tehran
[46]	Spain	Malaga	Natural parks, city parks
[47]	Benin	Abomey Calavi	Areas suitable for urban green spaces within the Abomey Calavi district
[48]	China	Beijing	Downtown area of Tongzhou District
[49]	Spain	Madrid and Guadalajara	Henares Corridor
[50]	Austria	Graz	Green Roofs in the City of Graz
[51]	China	Nanjing	The central city of Nanjing, including Xuanwu, Qinhuai, Jianye and Gulou districts
[52]	Ethiopia	Arba Minch	Different blocks of the city, parks, gullies and areas near major rivers
[53]	China	Xi’an	Fengdong New City of Xi’an
[54]	China	Suzhou	Tiger Hill

#### GIS data sources.

This review organizes and summarizes the GIS data sources for 20 articles. As shown in Table 4, they are divided into five categories: government organization, international organization, commercial company, academic institute, and other (open source community, web data , satellite data, etc.). The reasons are as follows: Government organization, these data sources are provided by national government agencies, which are usually authoritative and reliable; International organization, these data sources are provided by international organizations, which usually cover the global or transnational region and have wide applicability; Commercial company, these data sources are provided by commercial companies, which usually have high commercial application value and technical support; academic institute, these data sources are provided by academic institutions, which are usually used for scientific research and education; and other, these data include open source communities, satellite data and web data.

**Table 4:** Sources of GIS data for selected articles.

Study	Government organization	International organization	Commercial company	Academic institute	Other
[35]		✓	✓		
[36]	✓	✓			✓
[37]	✓	✓	✓	✓	
[38]	✓		✓	✓	
[39]	✓		✓	✓	
[40]		✓	✓	✓	
[41]	✓	✓		✓	✓
[42]	✓		✓		
[43]		✓	✓		
[44]	✓		✓		
[45]	✓				
[46]				✓	
[47]	✓			✓	✓
[48]	✓		✓		
[49]	✓	✓		✓	
[50]	✓				✓
[51]	✓		✓		✓
[52]	✓		✓	✓	✓
[53]	✓	✓		✓	✓
[54]	✓		✓	✓	

#### Types of UGI.

Table 5 presents a summary and conclusion of the UGI discussed in the 20 papers reviewed. These areas are classified as Parks and Gardens, Green spaces and forests, Waters and wetlands, Ecological corridors, and Special purpose areas for specific purposes. Parks and gardens serve as recreational and amenity facilities. Green spaces and forests offer ecological services, enhance air quality, and promote biodiversity. Waters and wetlands contribute to water resource management and ecological conservation. Ecological corridors connect various ecological zones and facilitate species migration and ecological balance. Lastly, there are special purpose areas. Ecological corridors are pathways that link distinct ecological regions, facilitating the movement of species and maintaining ecological equilibrium. Special purpose areas, on the other hand, serve specialized purposes and have designated management and conservation needs.

**Table 5:** Types of UGI for selected articles.

Study	Parks and Gardens	Green spaces and forests	Waters and wetlands	Ecological corridor	Special purpose areas
[35]	✓	✓			✓
[36]	✓	✓			✓
[37]		✓			
[38]	✓				
[39]		✓			
[40]	✓	✓			
[41]		✓	✓		
[42]		✓			
[43]	✓	✓			
[44]	✓				
[45]	✓	✓			
[46]	✓	✓	✓	✓	
[47]		✓			
[48]	✓	✓		✓	
[49]				✓	
[50]		✓			
[51]	✓				
[52]	✓	✓			
[53]	✓	✓	✓		
[54]	✓	✓			

#### Research questions and objectives.

The review of the selected 20 publications identified several study issues and aims, which are presented in Table 6. The review is divided into three main parts. The first part assesses the effectiveness of current UGI, including their distribution and coverage [35,44], accessibility [36,51], ecological benefits [39,50], and societal benefits [43]. The second part evaluates the potential effectiveness of UGI, covering appropriateness assessment [37,40,41,52], connectivity [46,48], and urban planning and design [38,49,54]. The third part explores how GIS can be used in UGI research, including decision support systems [42,53], models and tools [45], and data processing and analysis [47].

**Table 6:** Research content of selected articles.

Categorization	Study	Subcategory
Assessing the current effectiveness of UGI	[35] [44]	Distribution and coverage
	[36] [51]	Accessibility
	[39] [50]	Ecological benefit
	[43]	Social benefit
Assessing the potential effectiveness of UGI	[37] [40] [41] [52]	Appropriateness assessment
	[46] [48]	Connectivity
	[38] [49] [54]	Planning and design
How to use GIS in UGI research	[42] [53]	Decision support systems
	[45]	Models and tools
	[47]	Data processing and analysis

### GIS-based UGI assessment methodology

#### Calculation and statistics of intraregional indices.

Using GIS, a thorough and detailed evaluation of UGI was conducted by including several technological tools and data sources, based on these 20 literature references. One study employed the ArcGIS Spatial Analyst Toolbox to manipulate satellite image data [35]. This involved tasks such as image correction, identification, and information matching. The goal was to precisely extract the distribution of values for specific vegetation indices, such as Normalised Vegetation Index (NDVI). This approach not only measures the growth state of vegetation, but also establishes a strong basis for evaluating the quality of park green space. Furthermore, the researcher conducted on-site investigations and compared them with high-definition images to ensure more accurate calculations of accessibility [38]. This analysis yielded crucial data to support planning and management efforts. The application of GIS allows for the consolidation of important data regarding the park, including its total area, green area, plant species and quantity [43]. By combining this information with community population data, the per capita green space area can be calculated using statistical techniques [44]. These methods scientifically evaluate the availability and demand for green space resources, providing a deeper understanding of the current state of regional green spaces. For carbon stock estimation, GIS utilized photogrammetric data and onsite validation findings to gather information about buildings, plants, and other geographic entities using advanced image recognition techniques. In addition, [39] utilized the GIS platform to convert green roofs, gardens, and woodland areas into land use maps. They then estimated the carbon stock on each pixel using the InVEST model, which provided insights into the carbon capture capacity of the urban green space system. Moreover, they visually depicted the carbon capture and storage through spatial distribution maps.

Furthermore, the literature employs a comprehensive analytical approach that incorporates several sources of data, such as satellite photos, mobile phone GPS positioning data, and questionnaire survey results. This approach aims to provide a thorough evaluation of the functions and characteristics of urban green spaces. A comprehensive land cover map was generated by constructing a dataset on the GEE platform utilizing indices such as NDVI and satellite image data, and subsequently classified using support vector machine [44]. Subsequently, the data underwent a thorough analysis using ArcGIS. As a result, precise recommendations were made to improve the functionality and structure of green spaces, offering substantial assistance in optimizing and managing urban green space systems.

#### Analysing spatial distance and travel.

The Network Analyst module of ArcGIS is crucial in evaluating the availability and spatial arrangement of urban green spaces. The accessibility indicators at various geographic distances are determined by replicating the physical process of residents walking or driving to reach the green space [36,51]. This analysis takes into account not just the direct distances, but also includes a cost-distance analysis to more accurately represent the cost of transportation during walking and driving. The examination of accessibility using the Pathways Network provides a more detailed understanding of the variations in accessibility across different routes [49]. This analysis serves as a scientific foundation for improving the arrangement of green spaces [45]. To thoroughly evaluate the accessibility of green spaces, [40] computed the distances between green areas and essential facilities such as water lines and roadways. The pavement data was spatially queried to identify pavements that have adequate width. These pavements were then selected to obtain precise location information for implementing greening strategies, such as planting trees along the roadside [42]. Furthermore, the researchers examined the level of consistency and disintegration of spatial components. They employed GIS and GuidosToolBox (GTB) to identify possible ecological corridors. These findings served as valuable guidelines for developing the green network system [46].

During the thorough analysis stage, [49] utilized the Ordered Weighted Average (OWA) approach to merge the outcomes of several analytical methods (such as factor assessment and mapping, ecosystem service assessment, ecological connectivity mapping, etc.) in order to produce different UGI options. This method not only showcases the efficacy of GIS in data processing and analysis, but also highlights the possibilities of integrating GIS with other analytical applications, such as Decision Super. The CoCoSo (Combined Compromise Solution) method was employed in GIS software to evaluate the accessibility of green spaces to residents in each location [45]. Additionally, a decision matrix was created. An optimized solution for the distribution and accessibility of green spaces can be determined by requesting experts to rate and utilizing the Analytical Hierarchy Process (AHP) in Decision Super software. This research examines the utilization of internal GIS functions and the integration of GIS with external analysis tools. This integration offers scientific decision support for the development and administration of urban green space systems.

#### Generation of reference maps for decision-making.

Generating decision-making reference maps is a critical work in the diverse field of GIS applications. This effort involves combining the outcomes of intricate data analysis with precise geographical information to create a map representation that is both easy to comprehend and intuitively presented. Suitability maps are a crucial decision support tool that evaluate the appropriateness of a specific location for a particular purpose, such as constructing urban green spaces, protecting the environment, or planning agricultural layouts. These maps consider several environmental, social, and economic variables to make their assessments. The study investigates the problem of selecting acceptable locations for GI by gathering data from RS and use supervised classification techniques to reliably identify land use and cover types that are appropriate for urban green spaces [52]. Simultaneously, a comprehensive slope suitability map was created by integrating Digital Elevation Model (DEM) data, providing additional insights into the impact of topography on the building of green spaces. The AHP was employed to provide weights to the criteria, ensuring the impartiality and scientific rigor of the assessment outcomes. Using the GIS platform, a map of the spatial suitability of urban green space is developed by weighted overlay analysis. This map provides decision-makers with a precise reference for selecting suitable sites. The spatial analytic toolbox of GIS is used to collect diverse information, such as topography, rainfall, land cover, population distribution, and expert opinions. This information is then used for preprocessing and indepth studies, which involve collecting and analyzing data at the coordinates [40,47,49]. During the weight allocation session, they utilized multi-criteria analysis approaches, such as AHP, to guarantee the appropriate weighting of each aspect in the assessment process. Ultimately, the weighted overlay function in GIS combines the suitability maps of each factor with their respective weights to create a comprehensive suitability map. This map serves as a valuable tool for urban planning and improving green spaces, providing a scientific foundation and decision-making assistance.

#### Pre-analysis using GIS.

Prior to utilizing GIS for intricate spatial analyses, a sequence of meticulously planned preparatory steps is crucial to guarantee a seamless analysis procedure and yield precise and dependable results. Data pre-processing is an essential initial stage in GIS analysis. It encompasses the tasks of cleaning, organizing, transforming, and upgrading raw data to guarantee its quality and suitability for use. While this specific task may not be exclusively performed using GIS software, GIS tools are typically employed for tasks such as geographic data format conversion, coordinate system standardization, projection transformation, and other related processes. Furthermore, it may encompass image recognition [37,48], image classification [44,53], efficient information extraction [43], and the removal of extraneous influencing factors [35], among other tasks, to enhance the dataset for subsequent analysis. Field visits and research serve as vital supplementary steps prior to GIS analysis, as they validate the authenticity and accuracy of spatial data and provide detailed site information that cannot be directly obtained from RS data. Additional site features that are not accessible by RS data. Researchers can use field visits to confirm the precision of the spatial location, boundary delineation, and attribute information of satellite images, topographic maps, and other data [38]. This is particularly important for specific analysis targets, such as green roofs or roadside tree planting, as it helps determine the suitable location characteristics for these activities before conducting GIS analysis. To accomplish this, the researcher must initially define the specific characteristics needed for the analysis target, such as the age of the building, shape of the roof, and width of the pavement. Then, they can employ the spatial query and screening functions of GIS to extract the spatial entities that meet these conditions from the preprocessed dataset. This process not only facilitates the refinement of the analysis and enhances its efficiency, but also guarantees the pertinence and applicability of the results [42]. In GIS analysis, it is typically imperative to integrate datasets from various sources in order to conduct a thorough and unified analysis. Hence, following the completion of data preprocessing and field research, it is necessary to do data integration and standardization. This involves transforming data from various sources and formats into a format that can be recognized by GIS. It also involves harmonizing coordinate systems and projection methods, as well as carrying out data standardization (such as normalization and standardization) to enable effective comparison and analysis of different datasets.

Once the initial tasks are finished, it is necessary to create a comprehensive strategy and framework for GIS analysis. This should be based on the objectives of the study and the specific characteristics of the data. This encompasses the identification of the analysis procedures, the choice of analysis techniques (such as multi-criteria decision analysis, spatial statistical analysis, network analysis, etc.), and the establishment of the analysis parameters. Simultaneously, it is imperative to establish the manner in which the analytic results are presented, such as through charts, maps, and other visual aids. This is done to offer decision makers reference information that is intuitive and easily comprehensible.

#### Validation methods after analyses using GIS.

When the GIS analyses are finished, it is necessary to employ a range of validation techniques to evaluate the soundness of the studies and guarantee the precision and dependability of the results. These methods encompass a broad spectrum of characteristics, including data comparison, on-site validation, and subjective research, all aimed at ensuring the scientific validity and usefulness of the analytical findings.

A commonly used validation method involves verifying the accuracy of the analysis results by comparing them with known reliable data in GIS analysis. One way to confirm the accuracy of the proposed algorithms in finding appropriate sites is by comparing them with satellite pictures [42]. This approach utilizes the superior accuracy and real time nature of satellite imagery to spatially calibrate the findings of GIS analysis, hence ensuring the precision and consistency of the analysis outcomes. On-site validation is a crucial component of the post analysis validation process for GIS. Researchers must physically visit the analysis region in order to perform an on-site survey and compare the analysis results [47]. By doing field measurements, observations, and analyses, the precision of GIS analysis outcomes may be visually evaluated, potential issues and inaccuracies can be identified, and appropriate repairs and changes can be implemented. Field validation serves to bolster the credibility of analysis results and offers robust support for subsequent decision-making and planning. Subjective research and questionnaire validation are crucial methods for validating GIS through post-analysis. The process evaluates the rationality and suitability of GIS analysis outcomes by the gathering and examination of subjective viewpoints and input from the general public, specialists, or individuals with a vested interest [36,42]. This approach can compensate for the limitations of GIS analysis regarding human and social elements, hence enhancing the comprehensiveness and usefulness of the analysis outcomes.

Furthermore, the correctness and reliability of the analysis results can be further confirmed by comparing the consistency between the subjective study findings and the GIS analysis results. The determination of weights in multi-criteria analysis, such as the AHP technique, significantly influences the results of the study. Hence, ensuring the rationality and precision of weight allocation is also a crucial aspect of post analysis validation in GIS. This can be accomplished by thoroughly evaluating the process of assigning weights, comparing it with the opinions of experts, and AHP [47]. By employing the multi-criteria analysis method to validate weights, the findings of GIS analyses can be ensured to yield reasonable conclusions by taking into account many elements in a complete manner. GIS analysis relies on suitability ranking to evaluate the degree to which certain regions or objects are appropriate for a specific purpose or development. To guarantee the precision of the appropriateness ranking, it is vital to conduct essential validation work. This involves thoroughly examining the appropriateness ranking of the factors, for example, elevation and proximity to road network [47]. Based on this, necessary adjustments and optimizations can be made. Validating the appropriateness rating guarantees that the outcomes of GIS analysis offer robust support for subsequent decision-making and planning.

#### The change of GIS-based UGI in methodology.

The change of GIS-based UGI in methodology is reflected in three main areas.

Firstly, the methodology has become more refined and integrated. In 2020, researchers primarily used multi-criteria evaluation (MCE) techniques, such as the ordered weighted average (OWA) method, to conduct suitability assessments [49]. AHP and Multi-Criteria Decision-Making Analysis (MCDM) methods have emerged in the 2021 research literature [52], which enable a more refined and integrated assessment of the weights and impacts of different factors.

Secondly, visualization has become an integral component of the analysis method, presenting more comprehensive and detailed results. In early studies, the results of GIS-based suitability assessments were typically presented as simple maps. As technology has evolved, researchers have increasingly employed more sophisticated visualization tools to present assessment results. [51] utilized GIS software to visualize the analysis results, highlighting the distribution of service pressures, accessibility, and demand for green spaces in parks.

Thirdly, the interdisciplinarity of the methodology has been significantly enhanced. In recent years, GIS-based UGI assessments have increasingly incorporated an interdisciplinary knowledge system. For example, [53] integrated ecology, urban planning, and sociology to construct an urban green space ecological network by combining ecosystem functions with residents’ needs. This interdisciplinary assessment fully integrates socio-economic factors, thereby providing a more comprehensive picture of urban development needs.

In summary, from 2019 to 2024, GIS-based UGI methodologies have become increasingly refined. By visualizing the analysis, researchers can intuitively observe richer and more detailed analysis results. Interdisciplinary knowledge systems have increasingly been integrated into GIS-based UGI analysis and evaluation. These advances have not only enhanced the accuracy and efficiency of the analyses but also provided stronger support for urban planning, environmental protection, and sustainable development.

### GIS-based UGI assessment objects

This evaluation categorizes the objects of GIS-based UGI assessment into seven distinct groups for the 20 papers analyzed. According to the data presented in Table 7, they are Green space assessment evaluates the presence, purpose, and excellence of green spaces, encompassing factors such as dynamic alterations, extent, and health condition. Spatial analysis examines the arrangement and utilization of green areas and facilities to enhance their layout and usage, taking into account their spatial distribution and accessibility. Conducting an evaluation of the environmental and social advantages, which involves examining the overall benefits of green spaces to both society and the environment. Data management and decision-making include considering factors such as fairness, social effects, and environmental benefits. The topics covered include data management, decision-making, data gathering, and the use of analytical techniques for decision-making. These methods include criteria evaluation, multi-criteria decision analysis, and sensitivity analysis. Spatial modeling and analysis involve the use of spatial data to evaluate the state of green spaces and the environment, as well as to assist in spatial planning and analysis. Conducting visual and aesthetic evaluations to analyze the visual impact and aesthetic worth of green spaces and urban landscapes. Enhancing the visual experience by utilizing techniques such as visual field analysis and visual preference surveys. Additionally, performing environmental assessments to evaluate the visual impact of green spaces and urban landscapes, and optimizing the visual experience through similar methods. Environmental and ecological assessment refers to the evaluation of the ecological function, health, and environmental impact of green spaces. The researchers evaluate the role of green spaces in promoting ecosystem services, biodiversity, ecological connectedness, and other related features. They also analyze the influence of these characteristics on the environment.

**Table 7:** Categorisation of the selected articles for assessment.

Study	Green space assessment	Spatial analysis	Environmental and social benefits assessment	Data management and decision-making	Spatial modeling and analysis	Visual and aesthetic assessment	Environmental and ecological assessment
[35]	✓						
[36]		✓	✓				
[37]			✓	✓			
[38]	✓						
[39]		✓					✓
[40]		✓					
[41]			✓				
[42]		✓		✓	✓		
[43]	✓						
[44]	✓						
[45]		✓					
[46]		✓		✓			
[47]	✓						
[48]					✓		✓
[49]							✓
[50]							✓
[51]							✓
[52]		✓					
[53]							✓
[54]						✓	

#### Distribution and change of green spaces.

Urban green space planning and management techniques are gaining increasing attention in both academics and practice due to their role as essential providers of ecological services and their ability to enhance the well-being of people. [43] exposes a deficiency in local green space resources, underscoring the unequal distribution of green space and underscoring the essential nature of green space in promoting recreation and controlling the city’s temperature. [47] employed GIS and multi-criteria analysis to ascertain appropriate locations for urban green spaces. This involved considering environmental, geographic, and socioeconomic aspects, resulting in the provision of reliable data and scientific recommendations for green space design. [44] utilized satellite imagery analysis along with sophisticated algorithms to not only classify and evaluate urban green spaces, but also to detect the beneficial effects of green spaces on public health and well-being throughout the epidemic. [35] employed RS methodologies, including NDVI and GIS, to elucidate the fluctuating patterns of alterations in green areas and the associated environmental hazards. Their findings serve as a cautionary signal regarding the potential risks posed to the ecological integrity of green spaces due to industrial activity. [38] based on the GIS platform, the quantity, quality and accessibility of parks and green spaces in the central city were systematically assessed, and an optimal layout strategy was proposed, aiming to promote the fair distribution and efficient use of green space resources by increasing the supply of green space and improving the layout.

#### Spatial layout of UGI in the city.

Spatial analysis has played a crucial role in urban planning and environmental management by effectively utilizing its advanced data analytic capabilities to optimize the accessibility and layout of urban green spaces. [40] utilized GIS and AHP-MCDM approach to assess the suitability of green infrastructures in the region. This assessment considers not only natural variables such as topography and slope, but also emphasizes the scientific and rational aspects of green space development. [39] conducted a thorough analysis of the impact of urban greening on carbon storage using GIS and the InVEST model. Their findings highlight the significant significance of urban greening in mitigating climate change. [45] highlighted the unequal allocation of green spaces in Tehran, resulting in varying levels of accessibility for inhabitants. This underscores the importance of including spatial justice into urban planning for green spaces. [36] used a GIS network analysis tool to measure how inhabitants perceive urban green spaces. They assessed the accessibility of green spaces for residents who walk or drive, and provided statistics to help enhance the quality of urban life. [42] created GIS algorithm that integrates with a Computational Fluid Dynamics (CFD) model. This algorithm properly forecasts the beneficial effects of green roofs and roadside trees on urban ecosystems. The study highlights the significant potential of small scale green amenities in enhancing urban settings. [52] employed GIS and RS techniques to determine the most suitable site for urban green space expansion. This involved considering variables such as slope, land use, and other relevant criteria to guarantee the practicality and long term viability of the green space development plan. [46] employed GIS modelling and the MSPA technique to conduct a comprehensive analysis of the composition and interconnectedness of the GI in the Mediterranean city of Malaga. The objective was to offer a detailed assessment for the development of an environmentally connected and efficient GI inside the city. This study establishes a scientific foundation for the development of a connected and effective urban green space system that supports natural processes.

#### Social benefits.

While enjoying the many benefits of green space, there are also many challenges. [36] points out that although green space has a positive impact on residents in general, its distribution is often Green space scarcity in certain regions can be attributed to factors such as population density and economic development levels. Resources are few and insufficient to fulfill the demands of the population. [41] is a great reference for resolving this issue. A thorough evaluation of green space infrastructure was carried out using GIS and the AHP-based MCDA method, which helped identify the crucial elements that influence the appropriateness of green space. Furthermore, [37] introduced a framework that utilizes RS and GIS techniques to evaluate the appropriateness of extensive green roofs on a broad scale. This novel methodology offers us an original strategy for implementing GI. By utilizing building roof space for greenery, it is possible to not only expand the urban green space and raise the green covering rate, but also improve urban air quality and elevate the residents’ quality of life.

#### Visualization and aesthetics.

Visual and aesthetic evaluations are fundamental to urban planning and landscape design, and they significantly influence the comprehension and enhancement of the visual aspect of urban areas by decision-makers and designers. Visual Impact Assessment (VIA) is a commonly employed method for evaluating the potential alterations to the visual appeal of natural landscapes caused by urban development [55,56]. It specifically emphasizes the thorough examination of the visual aesthetic consequences on significant natural elements and landmarks. Quantitative indicators and techniques, such as computer-aided design [57], are commonly employed in landscape aesthetics assessment to offer planners and decision makers scientific insights into how various landscape layouts influence visual impressions.

#### Environment and ecology.

Current scholarly work emphasizes the utilization of GIS and MCDA to improve the evaluation of environmental and ecological aspects of ecological infrastructures, namely urban green spaces. In the Austrian city of Graz, [50] showed that the type of urban development and environmental conditions significantly influence the distribution of green roofs, revealing a strong link between the two and ecosystem services. For Spain, [49] proposed a GIS methodology to effectively design GI in the Madrid Guadalajara Henares corridor, emphasising its centrality in spatial planning. [41]. conducted a study in Taif Province, Saudi Arabia, where they used GIS and AHP to thoroughly evaluate the appropriateness of UGI. They specifically focused on the appeal of accessibility and visibility to the public for utilizing green spaces. In Fengdong New City, located in Xi’an, China, [53] utilized RS and GIS technologies to establish a green space ecological network. This innovative approach offers fresh insights for spatial planning.

## Discussion

The analysis results reveal that the GIS-based UGI assessment method encompasses both statistical and comprehensive calculations of the ecological and spatial data for a specific area. The utility of GIS extends beyond mere assessment; it also generates reference maps that inform decision-making processes, offering an intuitive framework for the planning of UGI. In the context of UGI assessment using GIS, the specific objects of evaluation are notably diverse [58]. The 20 articles selected for this study span a range of dimensions, including the spatial layout, social benefits, ecological environment, and visual aesthetics of UGI.

### What aspects of UGI can be assessed by GIS

GIS technology plays a crucial role in current urban planning and management by providing a unique and valuable tool for assessing and optimizing UGI. The utilization of this technology has a significant impact on the evaluation of UGI in various aspects, including ecological, society, economy, and health. Furthermore, it enhances the precision of assessment and planning.

GIS technology effectively uncovers the intricate correlation between the distribution of green space and the accessibility of residents, thanks to its robust spatial analytic capabilities. This analysis not only establishes a scientific foundation for optimizing the arrangement of green areas and enhancing service effectiveness, but also effectively advances the fairness of urban green spaces [41,51]. The distribution of vegetation in cities significantly impacts surface temperatures, with clustered vegetation proving more effective in reducing surface temperatures compared to scattered and fragmented vegetation [59]. It is important to emphasize that the implementation of GIS must be tightly integrated with the specific circumstances to ensure the precision and relevance of the approach in order to comprehensively capture the intricacy and subtleties of the real world scenario. The integration of GIS and RS technology enhances the efficiency and accuracy of monitoring urban green space coverage and greening quality [35]. This not only serves as a crucial point of reference for the creation and adaptation of urban greening plans, but also establishes a strong material basis for improving urban ecosystem services. Furthermore, the utilization of GIS in assessing the feasibility of rooftop greening has facilitated the extensive investigation of urban greening in a three dimensional manner. This has resulted in a more informed approach to increasing the quantity of urban greening, enhancing the microclimate conditions of buildings, and enhancing the ecological adaptability of the city. GIS technology utilizes MCDA to consider many elements such as topography, slope, humidity, and others [40,41,45]. This approach provides a reliable basis for determining the location, arrangement, and priority of green infrastructure, supported by scientific decision-making. This comprehensive analytical approach facilitates the equitable allocation and effective utilization of urban green areas, hence advancing the progression of sustainable urban development. GIS technology employs techniques such as least cost modeling and gravity models [48] to evaluate ecological corridors and connectivity. These methods help discover and prioritize green corridors inside urban green spaces, as well as assess the interaction and ecological significance of these corridors. This serves as a crucial foundation for constructing urban ecological networks and preserving biodiversity, while also offering scientific guidance for the formulation of strategies aimed at protecting and restoring urban ecosystems. GIS technology effectively evaluates the present arrangement, level of fragmentation, and demand for services of green areas by using various data sources such as satellite photos, NDVI, and POI (Point Of Interest) [43,51]. This data provides robust support for the scientific management and optimal distribution of green space, facilitating the logical allocation and effective utilization of urban green space resources. GIS technology offers a scientific approach to evaluating the ecological advantages of UGI, specifically by measuring the potential ecosystem services provided by GI like green roofs [50,53]. These services include regulating microclimates and improving air quality. GIS aids in the creation of an ecosystem service network for urban green spaces. It also assists in identifying crucial ecological corridors and nodes, offering valuable guidance for urban ecological conservation and restoration.

In the future, as GIS technology advances and its applications expand, its role in managing UGI will become increasingly prominent. To improve the evaluation and improvement of UGI, it is advisable to implement the Landscape Aesthetics Assessment for assessing visual perception preferences [60]. Additionally, the Stakeholder Driven Approach (SDA) can be used to develop a Green Infrastructure Spatial Planning (GISP) model that aims to maximize ecosystem services [61]. Given the escalating effects of climate change and the growing urban population, it is crucial to address and reduce the risk of flooding in urban sites [62]. UGI exhibits notable proficiency in functional metrics such as resilience, reliability, and sustainability of urban flooding and urban drainage systems [63]. GIS platforms are easily comprehensible for GI implementation, aiding in flood mitigation due to their substantial storage capacity, extensive spatial query flexibility, and streamlined decision-making process [64]. Furthermore, the presence of institutional and policy support is essential for the effective planning and execution of UGI. For example, New York has made significant investments in UGI with the aim of enhancing local environmental and socioeconomic circumstances [65]. Formulating policies to facilitate the involvement of UGI in spatial planning and terrestrial development, capitalizing on the favorable attitude of residents towards the significance of GIs in urban ecosystems [66–71], and recognizing that the public is the primary audience that values UGIs, along with conducting surveys to improve public perception and evaluation, will contribute to the advancement of sustainable development in UGI [72].

### How to use GIS for assessment

#### Multiple data feeds and integrated analyses.

To effectively utilize GIS for evaluation, it is crucial to integrate various data sources in order to conduct thorough spatial analysis. The primary datasets are satellite pictures [35,44,53], precipitation records, air quality data [37], demographic data [38], building plans, climatic functional zone maps, traffic noise levels [50], and POI data [51]. The presence of diverse data not only captures the intricate and multifaceted nature of the urban environment, but also enhances the comprehensiveness and accuracy of the evaluation results. Through the integration of this data, GIS may uncover concealed patterns and relationships within metropolitan areas, offering unparalleled understanding of urban planning and administration.

Given that various RS data (e.g., Landsat, LiDAR) can effectively monitor the fragmentation of green space and provide valuable references for its protection [73], the acquisition of satellite imagery is crucial for GIS-based assessments of UGI. Specifically, multispectral aerial photos can be utilized to compute crucial ecological indicators, such as NDVI, following the processes of correction, identification, and matching [35,53]. The comprehensive integration of these components not only enhances the precision of measuring the health condition of urban green spaces, but also improves the scientific rigor and applicability of urban planners’ decision-making process. Nevertheless, it is important to acknowledge that the accuracy and clarity of satellite image data significantly influence the outcomes of analysis. Therefore, it is crucial to exercise additional care during the stages of data selection and pre-processing.

The application cases of GIS in assessing urban green spaces are abundant and varied. These include evaluating the accessibility of park green spaces, assessing the quality of green spaces, identifying areas with potential for green roofs, and evaluating the accessibility and service demands of green spaces [37,38,50,51]. These applications showcase the immense capabilities of GIS in addressing urban environmental issues and offer a scientific foundation for enhancing the arrangement of urban green areas and enhancing the quality of urban ecosystems. Alternatively, the European concept offers a comprehensive approach [74] that combines the ecological service contribution, biodiversity, and connectivity components of GIS [75]. The abundant accessibility of open source datasets has significant promise for determining the quantity of studies, while the incorporation of tools in GIS will enhance analytical capability [76]. Nevertheless, it is important to acknowledge that the implementation of GIS assessment findings must be evaluated in light of the specific circumstances, and policies should be in place to guarantee the accuracy and utility of the results. While high-resolution imagery and open data policies have created new opportunities for urban green space research [77], it is crucial to recognize that the implementation of GIS assessment findings must be evaluated in light of specific circumstances. Policies should be in place to ensure the accuracy and utility of the results.

#### Inclusion of multiple analytical indicators and factors.

When utilizing GIS for assessment, it is crucial to incorporate multiple analytical indications and components. These indicators encompass several aspects of ecological and social factors. For instance, the carbon pool consists of four primary components: above ground biomass, below ground biomass, soil, and apoplastic organic matter [39]. When combined with GIS technology, these indicators can be analyzed by integrating data from several sources to visually represent the spatial distribution of carbon capture and storage. In addition, the planning and evaluation of GI involve more than just a collection of ecological indicators. It also considers social and economic aspects such as design suitability, opportunity cost [48], ecological connectivity, and ecological status [49], among others. GIS plays a crucial role in this process by incorporating remotely sensed data and employing advanced techniques like supervised classification and AHP for multi-criteria decision making [41]. MCDM approaches [41,52] enable the systematic evaluation and prioritization of various assessment criteria. This comprehensive evaluation method enhances the scientific rationality of the planning scheme and mitigates the bias resulting from subjective judgment. Furthermore, GIS effectively integrates the social characteristics of ecological units into the analysis framework, including factors such as people’ desire for outdoor leisure and the limited availability of ecological units. The approach of analyzing the integration of ecological and social requirements exemplifies the endeavors of GIS in achieving a balanced and mutually beneficial coexistence between humans and environment. By integrating sophisticated analytical techniques such BP neural networks, GIS can effectively measure the relationships between various elements, resulting in the creation of more unbiased and scientifically grounded decision support tools [53]. GIS enhances the spatial appropriateness analysis of green space by allowing planners to make more precise decisions. This is achieved by assigning weights to criteria such as slope, land use type, and distance from rivers, and then overlaying them [52].

GIS utilizes a wide range of analytical indicators and factors in the assessment process, showcasing its strong data processing and spatial analysis capabilities. Moreover, it plays a crucial role in promoting sustainable development and achieving a balance between ecological and social needs. For instance, while GIS can handle a variety of indicators, the complexity and interdependence of these indicators can sometimes lead to challenges in data integration and interpretation. Some innovations in evaluation indicators have not been rapidly replicated, which can hinder the widespread adoption of new and effective assessment methods. For example, although some studies have addressed the connectivity of urban green spaces, the LISA indicator (Local Indicators of Spatial Autocorrelation) has not been explicitly documented or utilized in the literature to assess the aggregation and dispersal characteristics of vegetation blocks [78]. As the volume of data and the complexity of analysis methods continue to increase, ensuring the scientific validity of analysis results while enhancing efficiency and reducing computational expenses will be a crucial consideration for the future advancement of GIS technology.

#### Subjective research aided by fieldwork.

Although GIS is highly effective in processing and analyzing data, the need of human involvement in assessing and designing urban green spaces should not be overlooked. [79] highlighted the significance of involving citizens in the process of environmental assessment. Subjective research and field surveys can be a valuable addition to GIS analyses. During a field assessment of nine parks in the city of Dohuk, the researcher documented the quantity and scientific nomenclature of trees, shrubs, climbers, and ornamental plants. This thorough field survey yielded comprehensive biodiversity data for the evaluation of green spaces [43] and also uncovered the concealed biological intricacy that lies beyond the GIS data. This procedure serves as a reminder that while GIS offers numerous advantages, it still requires proper guidance and verification by human beings to guarantee the thoroughness and precision of the analytical outcomes. Furthermore, the incorporation of expert evaluation added a level of qualitative complexity and professional viewpoint to the GIS analysis. By considering experts’ opinions and ratings, we can enhance the precision of measuring the impact of various land use/cover types on ecosystem services [49]. This approach, which integrates qualitative and quantitative analyses, allows GIS analyses to move beyond mere data stacking and become more realistic and informative. Expert appraisal not only improves the professionalism of GIS analysis, but also boosts its persuasiveness and credibility in practical application. In addition, the use of subjective research instruments like surveys enhances the humanistic implications of GIS analysis. The researcher effectively identified the best observation distance by gathering users’ visual preference data and integrating it with GIS cumulative field of view analysis [54]. This creative endeavor not only showcases the adaptability and variety of GIS technology in spatial analysis, but also highlights the significant influence of human experience and emotions in planning decisions. The Public Participation Geographic Information System (PPGIS) offers a means of evaluating the various advantages of UGI. This system establishes a connection between the aspects that are typically significant to individuals and those that hold importance to them in the physical environment. It specifically focuses on understanding individuals’ thoughts and emotions within the landscape [80].

While GIS technology is recognized for its robust data processing and spatial analytical capabilities, its complete impact cannot be fully achieved without the addition and assistance of human components such as field study, expert evaluation, and subjective research. The inclusion of human elements in GIS analysis not only enhances its scope and complexity, but also offers decision-makers a more thorough, compassionate, and pragmatic reference framework. Hence, in forthcoming urban planning endeavors, it is important to persist in investigating the amalgamation of GIS technology and human subjective study, alongside collaborating with researchers from diverse disciplines [81].

#### Enabling AI technology.

The utilization of AI technology in GIS offers robust assistance for data processing and analysis. The extensive integration of AI in the domain of GIS, particularly in the areas of image identification and classification, presents significant benefits. Automated processing capabilities of AI enable the rapid transformation of complicated data, such as satellite photos, into high quality information sources that can be analyzed [37,44,53]. This technique not only enhances speed, but also substantially minimizes human error, establishing a strong basis for later geographical analysis. Nevertheless, it is vital to consider methods for guaranteeing the openness and ability to track the handling of data with the extensive implementation of AI technology. Furthermore, the utilization of AI in determining the weights of assessment indicators represents a novel approach to the conventional assessment procedure. Intelligent algorithms, like the BP neural network, can eliminate the limitations of subjective judgment by engaging in autonomous learning. This allows them to give more objective and rational weights to the indications [53]. This modification not only enhances the scientific rigor and impartiality of the analysis outcomes, but also urges us to reassess the function and placement of human decision-making in data analysis. At the application level, the integration of AI and GIS has demonstrated significant resilience and effectiveness. The cases mentioned, such as multi-criteria decision analysis using image semantic extraction [37] and the use of support vector machines to classify satellite images of urban vegetation with high accuracy [82], generate land cover maps and perform comprehensive analyses [44], show that AI has a great potential to enhance the decision-making process and improve the quality of planning.

Geospatial Artificial Intelligence (GeoAI) is a cutting edge method that propels GIS into a new era [83]. GeoAI is a development strategy that utilizes advanced learning techniques such as Machine Learning and Deep Learning to offer efficient and inventive solutions for geospatial development [84]. The utilization of AI technology in GIS unquestionably presents unparalleled prospects and significant alterations to urban planning and management. In addition, it is imperative to maintain mental clarity and employ discerning analysis while consistently evaluating the constraints and potential hazards associated with AI technology. This is crucial to assure its optimal utilization in advancing and benefiting human society.

### Advances in assessment methodologies

Recently, the progress of GIS technology in UGI has not only expanded our assessment capacities but also promoted the research on analysis and planning of UGI.

Firstly, technical advancements have offered a more precise and scientifically rigorous data source for GIS assessment. The implementation of emerging technologies, such as wireless networked sensors [85], real-time monitoring [86], and satellite-based vegetation index representation approaches [87], can significantly aid in the preservation and identification of regular vegetation health. While hyperspectral classifications, such as NDVI, can provide information about habitat structure, evaluating species diversity and dispersal necessitates the involvement of citizen scientists to determine the existence of species [88,89]. The local green space percentage was determined successfully by integrating RS techniques with GIS [35]. Urban green space was accurately mapped using NDVI, and standardized estimation algorithms were suggested, resulting in significant enhancements in the accuracy and efficiency of the assessment. Nevertheless, despite the significance and advantages of UGI, the process of rapid urbanization leads to the depletion of natural landscapes and green spaces, which can have negative consequences for both the population and the environment [90]. Hence, to guarantee a fair allocation of urban green spaces, it is imperative to create and execute mechanisms for assessing and monitoring their changes. Some UGI could not be accurately measured due to limitations in the data sources. For instance, certain roofs with slight slopes can be utilized for green roof rehabilitation [91]. To get more precise measurements in the future, high precision LiDAR data [92] or inclined photogrammetry and modeling approaches can be employed.

Furthermore, GIS has expanded beyond the use of a single model and now incorporates several models and decision-making approaches. As an illustration, GIS was used in conjunction with the InVEST model to conduct a thorough analysis of the effects of urban greening on carbon storage and sequestration [39]. This analysis demonstrated the variations in the carbon cycle that occur under different land use and cover scenarios (LULC). The integration of many models in this method offers policy makers a more thorough foundation for decision-making. Meanwhile, [40] utilized a multi-criteria decision-making method called AHP-MCDM, which combined GIS with the AHP. The purpose was to create detailed suitability maps for local GI and determine the suitability levels of UGI in various regions. This study provides valuable support for regional planning. The implementation of deep learning algorithms has revitalized the use of GIS in evaluating GI. [37] introduced a framework that utilizes deep learning technology to evaluate the appropriateness of green roofs. They identified significant criteria using RS and GIS technology. The suitability of green roofs was measured by integrating the TOPSIS approach, and a sensitivity analysis was conducted. This approach enhances the cognitive aspect of the evaluation and also enhances the scientific validity and reliability of the assessment outcomes. The implementation of GIS technology has gotten more sophisticated in terms of spatial analysis and optimization. To effectively plan a UGI program and understand its ideal structure, it is crucial to know the specific locations of demand, supply, and connectivity services within urban areas [93]. There is often a discrepancy between the availability and demand for services in metropolitan areas [94]. Therefore, establishing measures that prioritize connectivity can help to close these gaps. Recent research highlights the significance of taking into account various spatial scales to understand how different policies are considered to be beneficial [94]. GIS is widely recognized as a crucial instrument for urban management and policymaking in order to attain greater resilience and sustainability in development [95,96].

Furthermore, GIS technology has made substantial advancements in the integration of multi-criteria assessments, analysis of ecological networks and interconnectedness, and evaluations of spatial justice and resident requirements. To create a well organized UGI strategy, it is essential to have a clear comprehension of the spatial distribution of demand, supply [93], and connection ecosystem service areas within metropolitan regions. Recently, there has been a growing focus on the idea of community needs based planning in GIS assessment. [52] utilized a multi-criteria method to forecast the optimal placement of urban green spaces. Additionally, [97] analyzed 15-minute community living circle, highlighting the significant correlation between green spaces and the daily lives of inhabitants. These studies not only demonstrate the idea of planning that prioritizes people, but also offer compelling evidence for improving the usefulness and convenience of urban green spaces.

The advancements in GIS technology have had a significant and wide-ranging impact on the assessment methodologies used for urban green amenities. These advancements have not only enhanced the precision, effectiveness, and comprehensiveness of assessment, but also stimulated the invention and progression of assessment techniques. As technology advances and applications increase, GIS will have an increasingly significant role in assessing UGI in the future.

### Limitations of the study

There are several limitations in this review that warrant acknowledgment and should be addressed in future studies. Firstly, among the 20 articles ultimately included in this study, we found that these research subjects did not include UGI in the Americas and Oceania, which resulted in an uneven geographical distribution in our research. Upon reflection and investigation, we considered that the main reason for this limitation was the restrictive application of screening criteria for keywords in the title or abstract. If the screening criteria for titles and abstracts were formulated after reading the full text of the papers obtained from the search, and after grasping the correspondence between the research content of the papers and words in their titles and abstracts, it would help to improve the comprehensiveness of the study. Secondly, our analysis is based on 20 academic articles published between January 1, 2020, and June 30, 2024. This timeframe may have inadvertently excluded some seminal papers from earlier years. While we have examined a number of empirical studies, particularly earlier seminal papers, might have been overlooked. Therefore, future research should consider expanding the data sources to enhance the comprehensiveness of the study. However, given that the objective of this paper was to analyze the latest trends and relevant publications on the use of GIS to assess UGI, we believe that these 20 papers were representative and comprehensive within the current research domain. It is unlikely that earlier publications would have significantly influenced our conclusions. Thirdly, owing to the substantial heterogeneity in the selected studies regarding methodology, data collection, sample size, and the level of detail in the presentation of results, we were unable to conduct a more in-depth meta-analysis. Lastly, due to the confidentiality of data in some articles, our study was unable to examine data management and practices in all reviewed studies. We advocate for improved data management and sharing practices to ensure confidentiality and ethical use of data.

## Conclusion

In order to reveal the methods and data for evaluating UGI using GIS from 2020 to 2024, our study used explicit literature inclusion and exclusion criteria to select literature. We documented and reported the process (S1 Table) and reason (S2 Table) for selecting literature. Finally, we selected 20 articles as research subjects. These studies contain diverse methods and data for evaluating UGI using GIS. For example, the sources of GIS data include government organizations, international organizations, commercial companies and academic institutes. The evaluation methodologies included multiple data feeds, integrated analysis with multiple indicators and factors, fieldwork-assisted subjective research, and the use of enabling AI technologies. These data types and methods reflect the research field’s focus on multi technology integration applications, emphasizing data and result validation [98], using advanced technologies for multidimensional analysis, and considering the research trends of social factors and residents’ needs [99].

For the purpose of enhancing the sustainability and livability of cities, it is crucial for urban areas to prioritize the creation of local green spaces that offer a wide range of functions. The versatility of UGI can contribute to various advantages such as climate change adaption [100], risk reduction, human welfare, and urban revitalization [101,102]. Similar to other technical approaches, this method necessitates a certain amount of data access and GIS expertise. To get optimal results, the planning strategy must not only be technically sound, but also prioritize transparency, participatory involvement, detailed analysis, and scenario decision-making [103]. When planning for UGI, it is important to consider its multiple functions and aim for a process that promotes connectedness, convergence, communication, and social inclusivity. This should be done with a focus on long term plans. with a focus on long term initiatives [102].

Our study conducted in our report includes an examination of previous UGI evaluations, as well as a thorough review of theories, models, and methodology. This analysis identifies the most frequently used methods and data models for assessing UGI, and high lights key elements that should be considered in future UGI development.

The present investigation offers useful insights into UGI conditions. Nevertheless, conducting future studies could significantly enhance comprehension of this field by implementing the subsequent recommendations.

Future research should focus on enhancing the precision and comprehensiveness of datasets, as well as ensuring the inclusiveness and dependability of data sources. By utilizing social media data and GIS technology, researchers can obtain detailed information about the distribution and demographic accessibility of green spaces. This will not only enhance our understanding of green space planning and design but also improve the scientific and predictive aspects of these processes [104]. Enhancing Public Participation and Perception Integration: Future research should aim to bolster the public participation mechanism by employing various methods such as questionnaires, visual preference tests, workshops, focus group discussions, and in-depth interviews. This will facilitate a comprehensive understanding and incorporation of the public’s viewpoints and preferences. This will not only improve the public’s acceptance of the project, but also ensure that the design of environmentally friendly facilities aligns more closely with the needs of people’s lives [105], thereby achieving a peaceful coexistence between humans and nature. Integration of multiple disciplines and thorough evaluation: An essential avenue for future research is the promotion of cross-disciplinary integration among urban planning, ecology, sociology, and economics. Through the development of a comprehensive assessment framework and the incorporation of experts from diverse disciplines, we can thoroughly evaluate the multifaceted advantages of urban green spaces, encompassing ecological, social, and economic benefits. Because these factors can have a significant impact on people’s physiology and psychology [106], we urge the government and decision-makers to implement proactive and supportive policies and laws to establish a strong institutional framework for the growth of UGI and the preservation of urban green space. Simultaneously, facilitate the advancement of data standardization and diversification to guarantee dependable data quality that is effortless to distribute and utilize.

Based on this review, we believe that researchers can enhance the GIS-based UGI assessments in the following three aspects. Firstly, the scope of GIS-based UGI assessments is expanding beyond traditional ecological domains to encompass social benefits and aesthetics. This trend indicates that GIS-based assessments can effectively address diverse aspects beyond ecology. Therefore, researchers can leverage GIS to analyze and solve more diverse problems in UGI from a broader perspective.

Secondly, while RS and other technologies are valuable for assessing UGI, many researchers still focus primarily on the ecological aspects of these facilities. However, many researchers are also paying attention to the actual living experiences of urban residents. The evaluation of UGI based on GIS is not limited to RS technology and GIS analysis tools, but also integrates data from RS, field research, and questionnaires. This reflects the human-centred research idea. Along this line, research in this field has the potential to explore more complex and diverse social issues related to UGI. By taking the life, health, and aesthetics of urban residents as the starting point and combining field research, questionnaires, and GIS analysis tools, research results can more profoundly reflect the problems of UGI and the real changes they bring to residents’ lives.

Thirdly, interdisciplinary technologies have been introduced into the research in this field in a large number of cases. In particular, AI technologies, such as image recognition and classification, continue to empower research in this field. With the continuous improvement of computational power and algorithms, knowledge discovery based on comprehensive geographic big data is expected to achieve breakthrough progress. Researchers should actively learn and apply AI technologies, integrating them with current GIS analysis methods to advance the field.

## Supporting information

S1 Table List of included and excluded references. (XLSX)

S2 Table Included references and detailed reasons for excluded references. (XLSX)

S3 Table PRSIMA 2020 checklist. (DOCX)

S4 Table Sample quality testing. (XLSX)
